# A Fog Computing Based Cyber-Physical System for the Automation of Pipe-Related Tasks in the Industry 4.0 Shipyard

**DOI:** 10.3390/s18061961

**Published:** 2018-06-17

**Authors:** Tiago M. Fernández-Caramés, Paula Fraga-Lamas, Manuel Suárez-Albela, Manuel A. Díaz-Bouza

**Affiliations:** 1 Unidade Mixta de Investigación Navantia-UDC, Universidade da Coruña, Edificio Talleres Tecnológicos, Mendizábal s/n, 15403 Ferrol, Spain; m.albela@udc.es; 2 Navantia S. A., Astillero Ría de Ferrol, Taxonera, s/n, 15403 Ferrol, Spain; mdiaz@navantia.es

**Keywords:** RFID, Industry 4.0, Shipyard, fog computing, identification, supply chain management, localization, tracking, cyber-physical system, IoT, IIoT

## Abstract

Pipes are one of the key elements in the construction of ships, which usually contain between 15,000 and 40,000 of them. This huge number, as well as the variety of processes that may be performed on a pipe, require rigorous identification, quality assessment and traceability. Traditionally, such tasks have been carried out by using manual procedures and following documentation on paper, which slows down the production processes and reduces the output of a pipe workshop. This article presents a system that allows for identifying and tracking the pipes of a ship through their construction cycle. For such a purpose, a fog computing architecture is proposed to extend cloud computing to the edge of the shipyard network. The system has been developed jointly by Navantia, one of the largest shipbuilders in the world, and the University of A Coruña (Spain), through a project that makes use of some of the latest Industry 4.0 technologies. Specifically, a Cyber-Physical System (CPS) is described, which uses active Radio Frequency Identification (RFID) tags to track pipes and detect relevant events. Furthermore, the CPS has been integrated and tested in conjunction with Siemens’ Manufacturing Execution System (MES) (Simatic IT). The experiments performed on the CPS show that, in the selected real-world scenarios, fog gateways respond faster than the tested cloud server, being such gateways are also able to process successfully more samples under high-load situations. In addition, under regular loads, fog gateways react between five and 481 times faster than the alternative cloud approach.

## 1. Introduction

Technology is evolving at a fast pace and companies have to adapt to such a constant evolution. In recent years, the industrial application of the paradigm of the Internet of Things (IoT) and the principles of Industry 4.0 have derived into the introduction of the latest technologies for monitoring, controlling and optimizing processes [1,2,3,4,5]. In this new industrial revolution, Navantia, a Spanish naval company that has been building hi-tech military and civil vessels for more than 300 years, decided that it was essential to adapt its inner workings to the Industry 4.0 principles to enhance its competitiveness. Such a decision led to the creation of the Navantia-University of A Coruña Joint Research Unit [6], a think tank aimed at creating a Shipyard 4.0: a modern shipyard that makes use of the latest technologies according to the Industry 4.0 principles.

**Figure 1:**
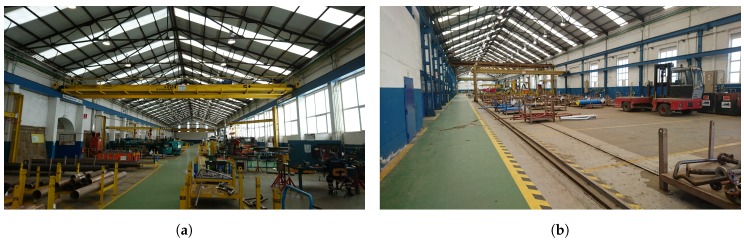
Left (**a**) and right (**b**) wings of the pipe workshop.

The Joint Research Unit deals with different parallel lines that work on heterogeneous topics like robotics, factory automation, process analysis or the application of new wireless communication systems on a ship. One of such research lines is called “Pipe Auto-ID” and, as its name implies, it deals with the problem of identifying pipes automatically through their life-cycle. Note that pipes are essential in shipbuilding: a ship usually contains between 15,000 and 40,000 pipes that differ greatly in their typology (i.e., size, material, shape, accessories), although they all are built at the same workshop in a shipyard that Navantia owns in Ferrol (Spain). The workshop is divided into two wings (shown in Figure 1), where pipes go through the different stages described later in Section 2.1.

In the previous work to this article [7,8], active and passive Ultra High Frequency (UHF) Radio Frequency IDentification (RFID) were selected and tested [9] in shipyard environments taking into account their security for Industry 4.0 applications [10,11,12]. Although the developed systems worked fine at a low scale, when the number of products to be tracked grew (and, as a consequence, the network traffic they generated), an obvious bottleneck was observed in the proposed cloud-based approach, which led to increasing latency responses and slower data processing. Among the different alternatives for tackling this issue, the design and implementation of a fog architecture was chosen, since a fog computing system is able to lower response latency, it provides location awareness and it is able to cope with a large number of wireless tags.

Therefore, this paper presents a fog computing based Cyber-Physical System (CPS) that makes use of active RFID technology to track pipes, to show information on them and, eventually, to detect and automate certain life-cycle events related to the pipes built in a shipyard. Thus, the main contribution of this work is the description, implementation and practical evaluation of a fog-computing architecture of a novel CPS for an industrial environment like a shipyard.

The remainder of this paper is organized as follows. Section 2 describes the life-cycle of a pipe in Navantia’s pipe workshop and reviews the most relevant traceability systems developed for shipyards as well as their main challenges. Section 3 details the design of the proposed system. In Section 4, the system is evaluated in different scenarios in order to determine its latency and its maximum data processing rates. Finally, Section 5 is devoted to the conclusions.

## 2. Related Work

### 2.1. Pipe Manufacturing in a Shipyard

**Figure 2:**
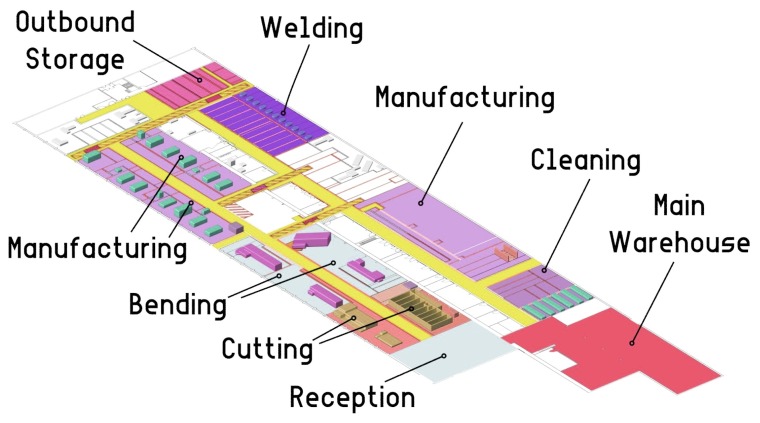
Navantia’s pipe workshop in Ferrol (Spain).

**Figure 3:**
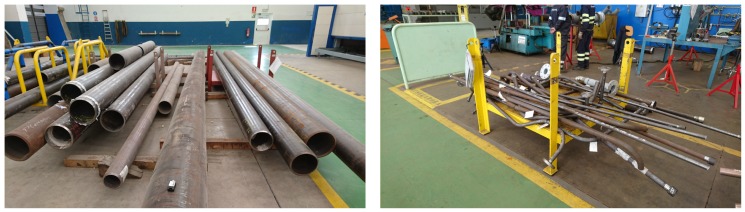
Reception area (**left**) and pipes stacked on a pallet after cutting (**right**).

The life-cycle of a ship pipe begins in the pipe workshop, whose floor map is represented in Figure 2. Initially, raw pipes are received from external providers and placed in the reception area (shown in Figure 3, on the left), where they are collected by operators according to production needs. The first processing stage occurs in the cutting area, where pipes are cut with high-precision saws. After cutting, a tag is attached to every pipe in order to identify it through its life-cycle. Traditionally, plastic labels with plain information, barcodes or QR codes have been used, nevertheless more sophisticated and human-centered approaches are emerging [13]. The system that is currently being deployed by Navantia is based on active RFID tags [7]. In this stage, pipes are stacked on pallets (like it is shown in Figure 3, on the right), which are moved throughout the workshop by using cranes.

**Figure 4:**
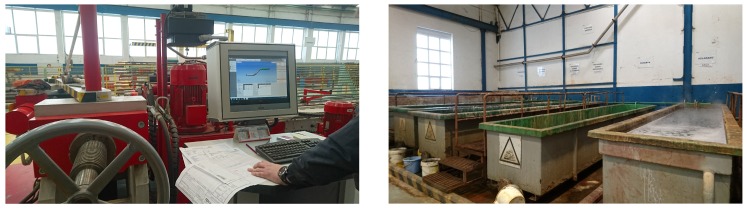
Computerized bending machine (**left**) and cleaning tubs of the pipe workshop (**right**).

The second stage for most pipes is bending (obviously, straight pipes do not need it). The procedure is performed with computerized bending machines (one of them is shown in Figure 4 on the left). Next, if a pipe requires to be cleaned chemically, it has to be moved to the degreasing and rinsing area, where acid and caustic solutions are applied in tubs (in Figure 4, on the right). After cleaning, pipes can go to one of the two manufacturing stages in order to add accessories (e.g., hydraulic valves, connection fittings), either by welding them or by using other techniques. Finally, the manufactured pipes are packed into pallets that are placed in the outbound storage area until they are required to be mounted in a ship.

**Figure 5:**
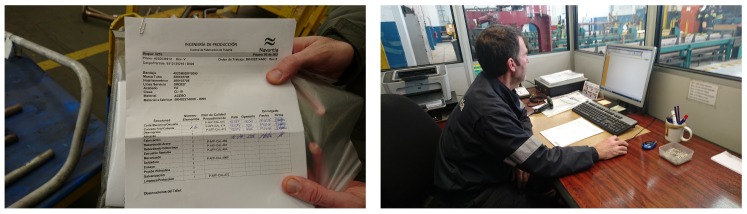
Traditional paper-based pipe process and quality controls.

It is worth noting that all the previously mentioned processes were traditionally carried out manually by Navantia’s operators. As it is shown in Figure 5, many processes made use of paper forms that, after being filled by the operators, had to be uploaded to the system by a supervisor, which clearly decreased the productivity of the workshop. Thanks to the CPS proposed in this paper, the vast majority of the information can be stored and modified through digital devices, removing most of the bottlenecks and errors related to manual fillings and uploads.

### 2.2. Potential Difficulties When Developing a CPS for a Shipyard Workshop

A shipyard workshop is in different aspects a tough scenario for deploying a CPS. Specifically, the main issues that can arise, are:

- High presence of metallic objects. As it can be observed in Figure 3 and Figure 4, the workshop contains many elements like pipes, pallets, machines, work benches or cranes, which are made out of metal. The problem arises when monitoring pipes or other elements of the workshop using electromagnetic propagation, which is influenced by the reflections created by the metal objects found in its path. This kind of signal interference is especially problematic for High-Frequency (HF) and higher radio frequency bands [8,14,15,16].
- High relative humidity levels. Shipyards are built next to the sea or to rivers, so relative humidity levels are usually high. In the case of Navantia’s pipe workshop, such levels oscillate throughout the year between 40% and 95%. Note that high levels of relative humidity may derive into problems with certain electronic devices. Moreover, in Navantia’s shipyard it is also common to find salt residues, since it is close to the sea and exposed to the action of the wind.
- Exposure to high temperatures. In certain areas of the workshop (i.e., in the welding, manufacturing and cleaning areas), pipes and some tools can be exposed to high temperatures.
- Presence of corrosive substances. In some areas of the pipe workshop (e.g., the cleaning area) it is common to make use of different acids, caustic solutions or fuel, which may condition the selection of sensors, actuators and other electronic devices.
- Presence of communication interference sources. The CPS communication architecture should take into account that there are in the workshop, besides common sources of electromagnetic interference (e.g., Wi-Fi networks or Bluetooth devices), other elements that generate electrical and electromagnetic noise. For instance, it is difficult to make use of Power-Line Communications (PLC) in the workshop due to the presence of mechanical saws and other AC-motor based tools that interfere remarkably with the communication through power lines. In addition, wireless communications can be interfered, for example, by the radar tests performed in the shipyard, whose power can reach several KW.
- Long communication distances. Most shipyard workshops are between 100 m and 250 m long, therefore, communications require the use of the proper technology. In the case of making use of wireless communications, it is almost certain that a network of devices or repeaters would be needed to cover a whole workshop. Moreover, network devices should be placed at spots with access to the data network and to electricity.
- Exposure to pressure sources. Like in other industries, in a shipyard workshop the products are moved in groups from one area to another, which usually leads to collisions and to the accumulation of weight on the products placed at the bottom. In the case of the pipe workshop, up to 35 pipes are commonly moved together in a pallet that withstands a weight of up to 2 T. Therefore, if sensors, actuators or other electronics are placed on the pipes or on the pallets, they should be protected with a proper encapsulation.

### 2.3. Shipyard Traceability and Cyber-Physical Systems

Ships are generally built using a construction method that divides them into several blocks [17,18,19]. Taking into consideration that many elements are built simultaneously, the different components that make up a block need to be managed at almost the same time across the shipyard. Furthermore, an accurate and efficient planning of the logistics among the different production areas of the shipyard (i.e., workshops, warehouses, stacking areas and docks) is a challenging task [20].

Traceability is a field that has evolved remarkably in the last years thanks to the progress associated with technology and standardization. In the case of shipbuilding, some authors proposed systems with the objective of keeping traceability of people and of diverse elements present in shipyards [21]. For instance, sensor networks have been introduced for monitoring different construction tasks [22,23]. Other authors [24] propose a Bluetooth system for positioning shipyard workers. The authors created a mockup workshop and tested the system, achieving a 1.2 m accuracy in a cluttered environment. A different approach is presented in [23], where the researchers detail a system that makes use of sensor networks and RFID to monitor processes and supplies in construction and assembly industries like shipbuilding.

With respect to cyber-physical systems, several challenges arise when they are applied to a shipyard or to other mission-critical infrastructures, since security, safety, trustworthiness, robustness, and interoperability are key aspects to be fulfilled for their broad adoption [25,26,27]. Due to this fact, just a couple of CPS for shipyards can be found in the literature [28,29]. One of them is aimed at monitoring vehicles [28], while the other one allows for supervising remote facilities and utilities in a shipyard [29].

Shipbuilders usually do not develop their own cyber-physical systems, but rely on different commercial software that, in some cases, is integrated into a common system. The latest trend consists in incorporating a Manufacturing Execution System (MES), whose aim is very similar to the one of an industrial CPS: to track, collect and show information on the products during the manufacturing processes. Companies like Siemens or SAP sell their own MES, which is adapted to the specific necessities of a field, factory or workshop. A MES usually has to be integrated with two additional pieces of software: the Enterprise Resource Planning (ERP) and the Product Life-cycle Management (PLM) software. This integration eases data sharing between the functional areas of engineering, workshops and the front office.

Finally, regarding the use of fog computing for cyber-physical systems, some examples can be found in the literature for smart manufacturing environments [30,31,32] and high-security applications [33], but it has been found that none adapted explicitly to the necessities of a shipyard workshop like the CPS proposed in this article.

## 3. System Design and Implementation

### 3.1. System Architecture

**Figure 6:**
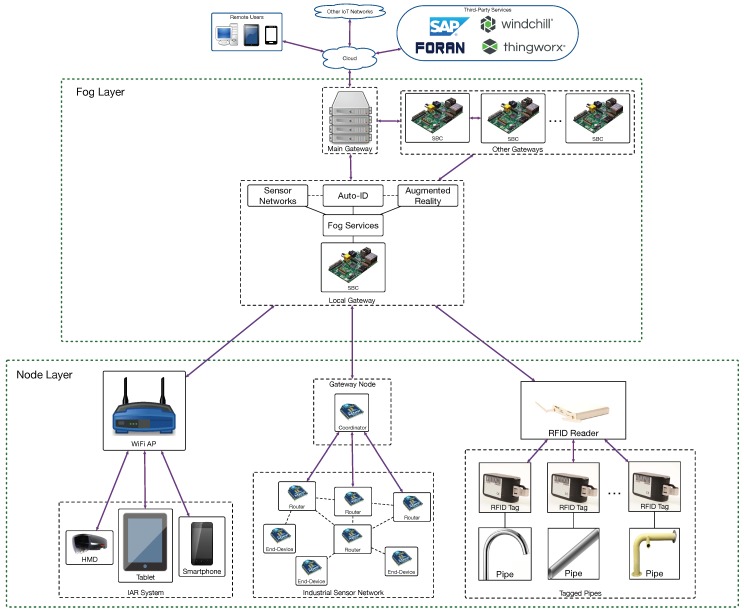
Fog-computing architecture of the proposed CPS.

The proposed system architecture is shown in Figure 6. As it can be observed, it is a three-layer fog computing architecture. The layer at the bottom is the node layer and includes all the devices (i.e., RFID devices, sensor networks and Industrial Augmented Reality (IAR) interfaces) that make use of the services provided by the fog layer. The fog layer is composed by Single-Board Computers (SBCs) that are scattered throughout the shipyard and that act as gateways. The SBCs provide fog services, including low-latency and data processing services like the RFID positioning service, sensor fusion, or data caching for streaming content to IAR devices [34,35]. Since this paper is focused on traceability, it will only detail the inner workings of the fog positioning service.

The internal cloud is in the top layer, where Navantia runs its own compute-intensive services and the ones offered through third-party software (basically: SAP as ERP, FORAN for ship design, Windchill as PLM, and ThingWorx as IoT platform).

### 3.2. Node Layer

At the workshop, active RFID devices, IAR interfaces and transducers from different sensor networks constitute the node layer. Regarding the RFID devices, there is a network of RFID readers that continuously collect data (basically, Signal Strength Indicator (SSI) readings) about the location of the pipes, which are controlled by a software system managed by the fog layer. Active RFID fixed readers [36] were selected due to the wide area to be covered in the workshop. In addition, active RFID tags [37], specially designed for harsh industrial environments, are used. Such tags, which operate at 433.92 MHz, can communicate with each other over distances of up to 100 m when using the appropriate antennas. Each tag reports its ID every two seconds for battery saving purposes.

### 3.3. Fog Layer and the Cloud

The indoor positioning system is managed by a fog service that is divided into different modules, the Location module and the Business Intelligence (BI) module being the most important (they are described later in Section 3.4.1). The Location module estimates the coordinates of the tags after processing the information gathered by the RFID readers. Then, the BI module detects events based on the movement of the pipes and decides whether to send notifications to other systems or clients. For example, one of such systems is the SAP connector, which is responsible for creating a communication link with the ERP and/or the MES, which are offered as third-party services by the cloud-computing layer. It is actually in such third-party systems where pipe information is stored.

### 3.4. Indoor Positioning Fog Service

The indoor positioning fog service was designed as a web application that involves two main components: a back-end and a front-end. The back-end is where all time and resource consuming tasks happen, while the front-end acts as a user interface, providing a way for operators to interact with the system.

The data managed by this fog service are generated by elements of the node layer (i.e., RFID tags and readers) and by the users that interact with the system. In the case of RFID readers, they constantly report tag SSI levels to the indoor positioning fog service. From the service perspective, the readers are just like clients that generate data through web requests at a constant rate. Note that, as the traceability system grows, the number of tags and readers increases, which implies that the number of requests to be handled also increases. In a cloud-centric approach, these increases might derive into traffic bottlenecks. Fortunately, the designed fog computing based architecture eases scalability as the indoor positioning system grows.

**Figure 7:**
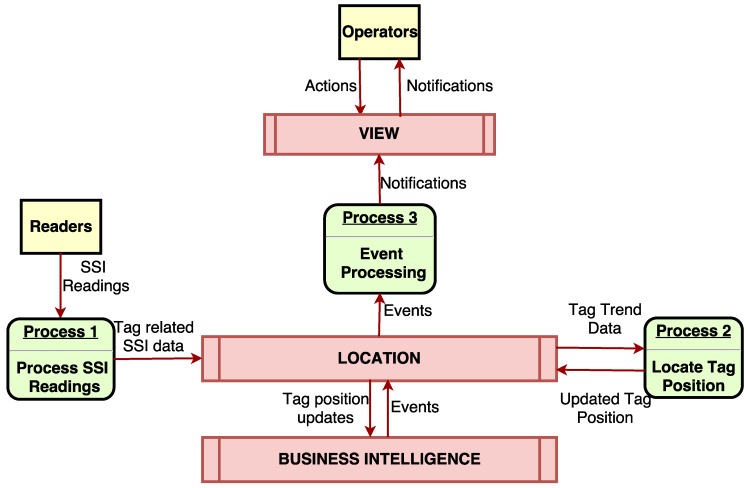
Communications in the indoor positioning fog service.

Figure 7 shows how the different sources of data from the node layer (i.e., readers and operators) interact with the indoor positioning fog service. Readers provide data to the fog service back-end in order to process them for the location and tracking algorithms. In the case of operators, they interact with the display module, providing certain data related to their tasks, mainly details of the pipes to be processed.

In Figure 7, Process 1 is the one in charge of obtaining the data from the readers. Since every reader embeds an HTTP server that must be queried periodically to obtain the SSI samples from the tags, these communications are performed through standard HTTP requests.

The samples obtained from all the readers need to be transmitted as fast as possible. When deploying in the same machine, both the process in charge of querying the readers and the location service, they communicate with each other by using Redis [38], an open-source in-memory data structure that allows for exchanging data between separate processes in a seamless and almost instant way. However, in a fog-computing or cloud-based architecture, a network mechanism must be used to communicate these two services, which run on different machines. Moreover, it must be taken into account the fact that fog gateways are resource-constrained, so only mechanisms that minimize computational requirements should be considered. Due to this reason, the execution of Redis directly on fog gateways was discarded.

Possible alternatives to the use of Redis would consist in sending the collected samples through standard HTTP POST requests or by using HTTP long polling, but since the number of requests per second is very high, they would introduce latency and overhead in both the network and the machines when sending and receiving the data. Finally, the chosen technology was web sockets [39], which are used for establishing long-term TCP connections between a client and a server, allowing for the creation of bi-directional full duplex communications channels. In addition, messages exchanged through web sockets are distributed instantly, with little overhead, resulting in very low latency connections.

#### 3.4.1. Location and BI Modules

As it can also be observed in Figure 7, the fog service back-end is divided into two modules: the location and the BI module. The location module receives data from RFID readers, processes them and determines each tag position in the workshop (the estimation of the position is explained below). Then, it sends the updated locations to the BI module, which is the one responsible for detecting and notifying pipe-related events. Four types of events are currently considered by the system:

- Area change events. When a pipe moves from one area to another (e.g., from the reception area to the cutting area) and it remains on the latter for a certain amount of time (configurable by the system), the event is captured by the system and shown to the operators that might be interested in it. This is especially useful for warning the operators about incoming work.
- Pipes leave the workshop towards an auxiliary company. When the workshop is overloaded, part of the procedures performed on pipes are outsourced to auxiliary companies. Such pipes are stacked on the outbound area of the workshop and leave it through a specific door that the trucks of the auxiliary companies go through to collect the pipes. In this situation, three events have to be detected. First, that some pipes have remained in the outbound stack area for a certain amount of time. Second, that the pipes include in their life-cycle a task that requires outsourcing. Third, that the pipes are no longer detected by the system readers either inside the workshop or in the neighboring dock.
- Pipes leave the workshop to go to another workshop or to be mounted on a ship. Similar to the previously described event, pipes are monitored by the system readers until they leave the workshop and then are detected again in a dock or in another workshop of the shipyard.
- Accessories are required for pipe manufacturing. When a pipe reaches the manufacturing area, in the case that it requires certain accessories to be added, it is automatically notified to the main warehouse, where the operators collect the accessories and carry them to the manufacturing area.

**Figure 8:**
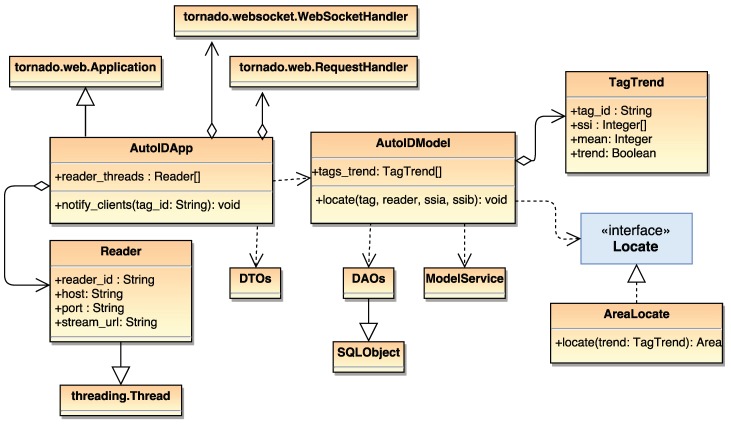
Location class diagram.

Figure 8 shows a simplified class diagram of the developed location subsystem, where it can be observed that Tornado [40] was used as the web server. With respect to the location algorithm, it is first important to note that pipes cannot be located anywhere in the workshop: there are specific areas where the different pipe processes are performed. This leads to the creation of logic areas, which are related to relevant physical areas of the workshop (e.g., storage areas, pipe processing machines, work benches). Every logic area is associated with an RFID reader and, thus, one or more readers cover a physical area.

For every tag, its position is associated with the RFID reader that receives the maximum power levels from the tag (i.e., it is assumed that the higher the power level, the shorter the distance to the tag). Therefore, the system compares in real time the power levels reported by all the readers and chooses the highest one. Then, since only one reader is associated to one logic area, it is possible to indicate in which logic area the pipe is most probably located.

The coarse estimations given by the location algorithm are refined in two ways to avoid false logic area detections and to correct ambiguous readings in the limits of the areas:

- The previous locations of a tag are considered when calculating its new position. Such a list of locations is called “tag trend” and consists in a set of data structures that store past SSI readings. Thus, the SSIs used for selecting the closest reader are conditioned by past readings. Therefore, if a tag has a $SSI_{t,i}$ at time instant *t* from reader *i*, the new $SSI_{t+1,i}$ can be computed as:
  $$SSI_{t+1,i}=\frac{SSI_{t,i}+\mu *ReceivedSSI_{t+1,i}}{2}$$
  where $ReceivedSSI_{t+1,i}$ is the actual SSI received at time instant t+1 from the reader *i* and μ is a speed conversion parameter that determines how fast the current SSI converges to the most recent value. Note that the use of μ slows down the convergence, but it allows for avoiding sudden changes in the SSI that occur from time to time due to signal interference (i.e., metal reflections or the presence of operators working). From our empirical experience in the workshop, a value of μ between 0.7 and 0.9 gives the best trade-off between convergence speed and oscillation avoidance.
- The way logic areas are defined involves a pair of SSI thresholds that limit the size of an area, letting the system adjust each area size independently. Thus, one threshold (called outer threshold) limits the total size of the area, while the other one (inner threshold) is set to indicate that the tag is really close to the reader. It is important to point out that, since logic areas are limited in size, blind spots exist (i.e., not every inch of the workshop is covered by a logic area). While this fact may seem a limitation, in practice, it adds a nice feature to the system, because, when all the possible pipe routes are covered by logic areas, it is straightforward to discover pipes that are located out of them (i.e., in a place where they should not be) and then warn operators about such inconsistencies.

**Figure 9:**
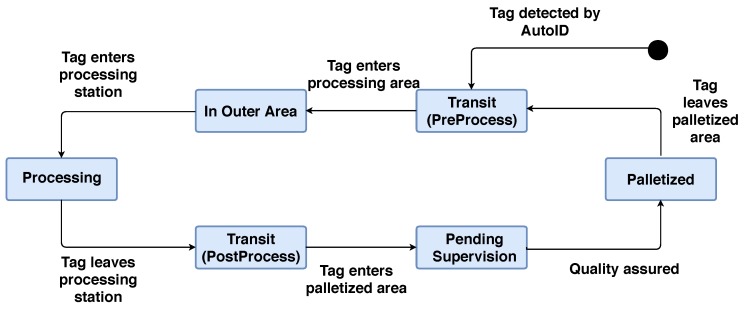
Pipe life-cycle state machine.

In addition to the previous refinements, in order to avoid positioning the pipes in wrong areas or showing the operator non-continuous positions (if the SSI oscillates heavily due to interference, the system might show the operator that a pipe is at time instant one in a logic area, while at instant two, it is dozens of meters away in another logic area), the life-cycle of every pipe is modeled according to the state machine diagram shown in Figure 9.

A pipe begins its life-cycle in the workshop when it is first detected by the RFID system. In that moment the pipe is considered to be in transit. Note that every pipe has to stop at every production area of the workshop, but the pipe can be processed or not in such an area. If there is no need to process the pipe (e.g., not all the pipes need to be bent), it is palletized (i.e., it is placed on a pallet). In the case that it needs further processing, then it first would have to be moved to the processing area, cross the outer area, be processed, and then leave the processing station towards a pallet where a quality control would be performed.

This capacity for being able to determine when a pipe is in a specific area or next to a production machine enables the automatic detection of pipe-related events. Specific events are notified when a pipe moves from a processing area to another, or when it crosses from an outer area to an inner area. Thus, the BI module receives periodic updates of pipe locations, processing them in conjunction with past locations and pipe production orders in order to determine the next state for the pipe, which is notified to the corresponding operator to indicate that a task will be added soon to his/her pool.

**Figure 10:**
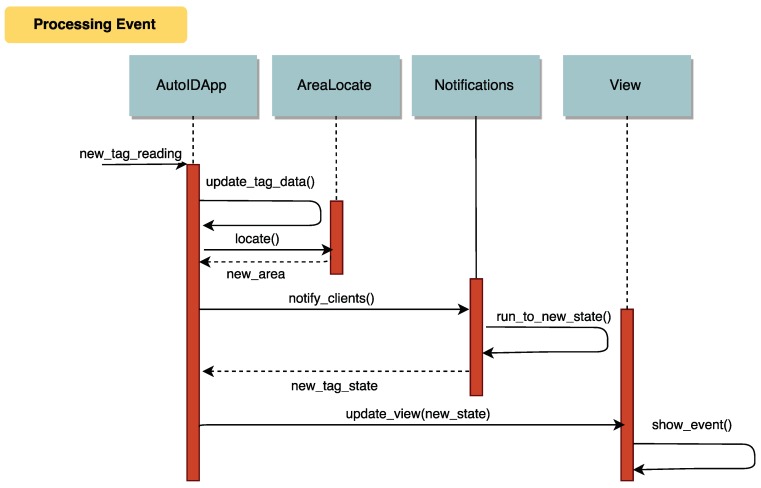
Generic notification sequence diagram.

A generic notification process is presented in the sequence diagram shown in Figure 10, which illustrates how the main components of the fog service interact through time. All the events are generated, detected, processed and notified automatically. The only verification process that is carried out manually is quality control, although, when a pipe is placed in a quality check area, an event is triggered to notify the new task to the quality supervisor, who can approve digitally (i.e., by using a tablet connected to the CPS) the quality check or disapprove it, indicating the detected problems.

#### 3.4.2. Display Module

**Figure 11:**
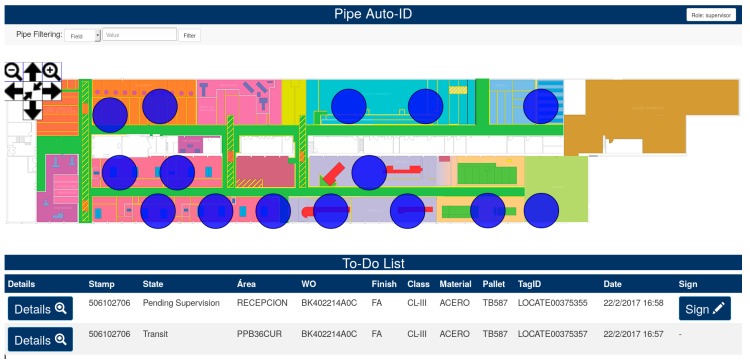
Display module interface.

The display module (in Figure 11) has been developed as a web application with the objective of being able to use it not only in specific software platforms, but in any system with a web browser installed (e.g., tablets, smartphones, desktop PCs, Macs). It consists of a map of the workshop where pipes are displayed together with a task list that indicates the pending work to the operators. Both components are updated in real time by the fog service. In the case of the task list, its updating depends on the area where the operator is located, since every operator also wears an active RFID tag (in a pocket or hanging from his/her belt).

**Figure 12:**
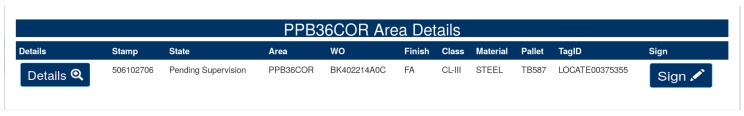
Details of the pipes located in a logic area.

**Figure 13:**
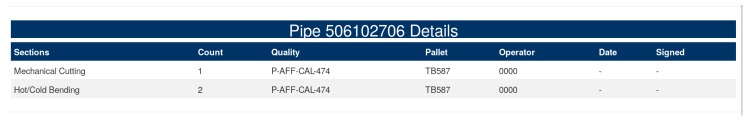
Details of a specific pipe.

Regarding the interaction, operators can zoom in and out of the map by using the control panel in the top-left corner of the canvas. In addition, logic areas (represented as blue circles) can be clicked or tapped, opening a new window that shows details on the pipes located in such an area (in Figure 12). The details of every pipe can be observed by clicking on its identifier (in Figure 13). Such details include the fabrication process that must be performed on a pipe, the area where the pipe is traveling to, a timestamp of every process already carried out, the state of the different quality check approvals and the quality procedure to be followed in every stage.

Note that the details shown about a pipe in an area depend on the role of the operator using the application. For example, only supervisors can make use of the sign button to approve a pipe quality control (in Figure 12, on the right).

**Figure 14:**
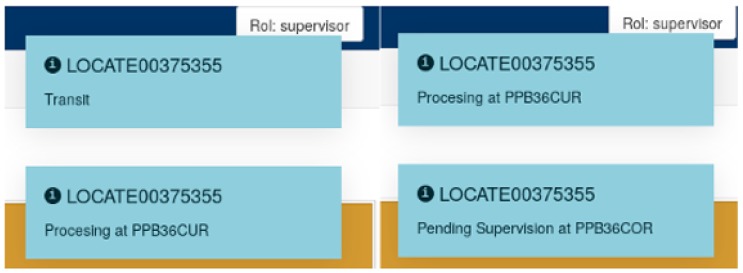
Event notifications on the display module.

With respect to event detections, they are notified in the display module visually through pop-up messages. As an example, Figure 14 shows different pop-ups that are displayed when a pipe goes through the different processes required since it is in the cutting area until it reaches the bending quality check area.

### 3.5. Integration with Third-Party Systems

To facilitate daily-basis operations at the pipe workshop, a MES was deployed in parallel with the pipe CPS. The MES accesses SAP ERP information about every Fabrication Order (FO) and displays the relevant data to the shipyard operators in a user-friendly way.

**Figure 15:**
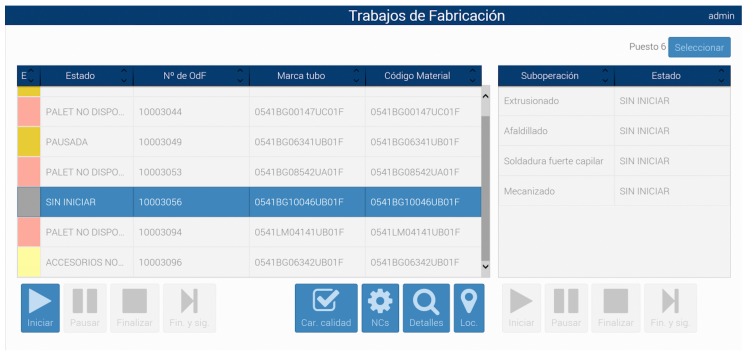
Screenshot of a manufacturing task pool of the MES.

The MES back-end was developed on top of Siemens’ Simatic IT [41]. The front-end consists in a web application designed to be accessed through tablets when wearing gloves. As an example, in Figure 15 such a front-end shows the task pool of one of the operators at the manufacturing stage. On the left, the system indicates for every task its state (*Estado*), the FO number (*N*° *de OdF*), the pipe ID (*Marca tubo*) and the pipe material ID (*Código Material*). On the right of the screen, the details on the different sub-operations to be carried out on the selected task are shown. At the bottom, diverse icons allow operators to begin, pause and stop the selected task/sub-operation. Other icons are related to show the quality characteristics of the task (*Car. Calidad*), to transmit to the engineering team the detected errors (*NC, No Conformidades*) or to show the position of the materials used in the task/sub-operation (by clicking on the *Loc* icon).

The ERP and MES group the individual tasks to be performed in FOs. A FO is composed by some materials (at least one pipe and, in some cases, one or more accessories) and a series of consecutive fabrication processes (e.g., cutting, bending, welding). Several actions regarding the FOs can be performed through the MES, such as starting or finishing FOs, starting or stopping fabrication processes, or outsourcing certain processes to auxiliary companies. Among the many possible actions, the following are the most relevant when integrating the MES and the pipe CPS:

- Tag assignment to pipes. An RFID tag can be assigned to a pipe or to a pipe accessory by using the MES. For such a purpose, first, an operator starts a cutting operation related to a FO in the MES. After the pipe is cut and ready to be palletized, an RFID tag is attached to the pipe. Then, the operator reads the tag ID with a reader, which is sent to the MES. Next, the MES notifies the event to the CPS, which stores the relationship pipe-RFID tag in its internal database and starts tracking the pipe.
- Detection of a tag when it enters or leaves a logic area. For instance, after completing a pipe cutting operation, the next operation could consist in welding the pipe to an accessory. In such a situation, when the fog service detects the pipe entering the welding area, it notifies the event to the MES, which updates the welding task pool and sends a warning to the welder to let him/her know that a new pipe is coming to the welding area.
- An operator requests the location of a pipe using the MES. In this case the MES sends the ID of the pipe to be located to the indoor positioning fog service. Then, the service queries its local database of active RFID tags and looks for the one with the required ID. In the case of finding it, the RFID tag location is returned to the MES, which presents a map to the operator that highlights the area where the RFID tag is located.

**Figure 16:**
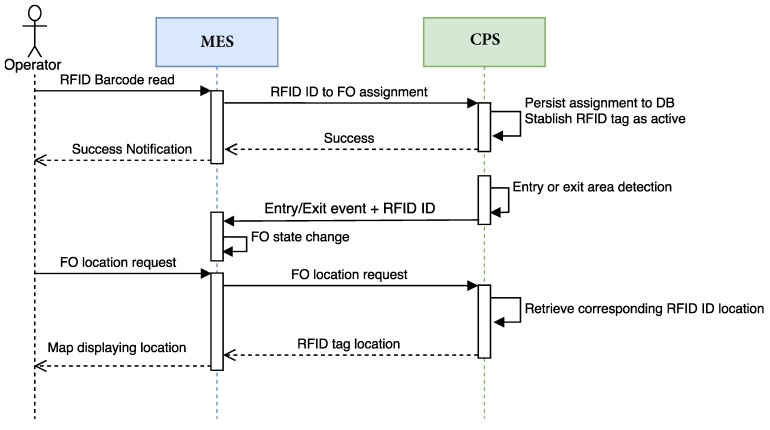
Sequence diagram of the interaction between CPS and MES.

Figure 16 shows a simplified sequence diagram that illustrates the three previous CPS-MES interactions. In the sequence diagram, it is assumed that an FO with only one pipe is processed.

## 4. Experiments

### 4.1. Experimental Setup

#### 4.1.1. Hardware

In order to evaluate the performance of the proposed architecture, it was tested when deploying the indoor positioning service either in the fog or in the cloud layer. In the case of the fog layer, the service was executed on an SBC that acted as gateway. Among the different SBCs that may be used (some of the latest and most relevant are compared in Table 1), the Orange Pi PC [42] was selected, since it provides a good trade-off between cost (as of writing, its price starts as low as $ 15) and features: it embeds a low power-consumption System-on-Chip (SoC) (a 1.6 GHz quad-core ARM Cortex-A7 Allwinner H3), 1 GB of RAM, Fast Ethernet, micro-SD slot, two USB 2.0 ports and an HDMI output. Regarding the cloud, the service was deployed on VMWare ESXi in a 64-bit Debian virtual machine with 4 GB of RAM that ran on two Intel i7-5500U@2.4 GHz cores.

#### 4.1.2. Software

To test the performance limits of the system, instead of relying on physical RFID tags and readers, their behavior was emulated through software processes that recreated the future Shipyard 4.0 environment, where thousands of tags will be monitored at the same time. Thus, every virtual tag, like every physical tag, sends its SSI every two seconds, which, depending on its position, is received by one or more virtual readers. The virtual readers make use of the same access interface as the real ones, allowing the reader subsystem to connect to them transparently and send the received SSI samples to the indoor location subsystem like in a real deployment.

Once the emulator subsystem is started, an algorithm is used to move the tags virtually around Navantia’s pipe workshop. A delay between two consecutive algorithm executions is randomly chosen from a uniformly distributed interval which goes from 0 to 0.01 s. With each execution of the algorithm, from all the available tags, one is selected randomly and its position is modified. Then, based on the new tag position, the received SSI value of each virtual reader is recalculated. This yields an average of 20 tags being moved every second.

Thirty virtual readers were created, since this is the number of readers actually deployed at Navantia’s pipe workshop. Every virtual reader requires an independent process that sends HTTP requests periodically, implementing the same randomly delayed approach used by the tag movement algorithm, but with a delay interval ranging from 0 to 1 second.

To manage the entire process easily, the virtual reader manager software needs a way to execute parallel code. Each virtual reader has to deploy an HTTP server that will be queried asynchronously, at very short time intervals. One approach could consist in using Python threads, by launching a thread for each virtual reader and then running a simple web server on each thread. This could be an option for a reduced number of threads, but due to Python GIL limitations [49], the performance of the virtual reader manager would be compromised when deploying several virtual readers. A better option is to use coroutines, the alternative recommended by Python for executing asynchronous code [50]. Since both simple web servers and coroutines were needed in the implementation, the virtual reader manager makes use of Tornado [40], a web framework and asynchronous networking library that simplifies the process of launching different webs servers by using coroutines.

Finally, to perform stress tests on the proposed system, an HTTP benchmarking tool was needed. There are a lot of applications aimed at benchmarking and stress testing HTTP servers, like Siege [51], Apache Benchmark [52], httperf [53] or WRK [54]. Among them, a tool was required to query the virtual readers in a simultaneous and asynchronous manner to represent a scenario as close as possible to the real one. For this reason, Locust [55] was the selected benchmarking tool. Locust is a load testing tool written in Python that allows for testing several HTTP endpoints at the same time. It allows for defining the number of simultaneous users to be emulated and the rate at which they are launched. Through a simple Python interface, the desired HTTP requests are generated, enabling to define the endpoints to be accessed and the way virtual users must behave (i.e., the delay between requests). Thus, when executing a specific Locust configuration, the virtual users are launched and the defined requests are randomly distributed among them, initiating the stress test and showing real-time statistics. Locust presents clear and meaningful statistics about the connections and also provides metrics about failed requests, median, average, minimum and maximum times, and requests per second.

#### 4.1.3. Experimental Scenarios

**Figure 17:**
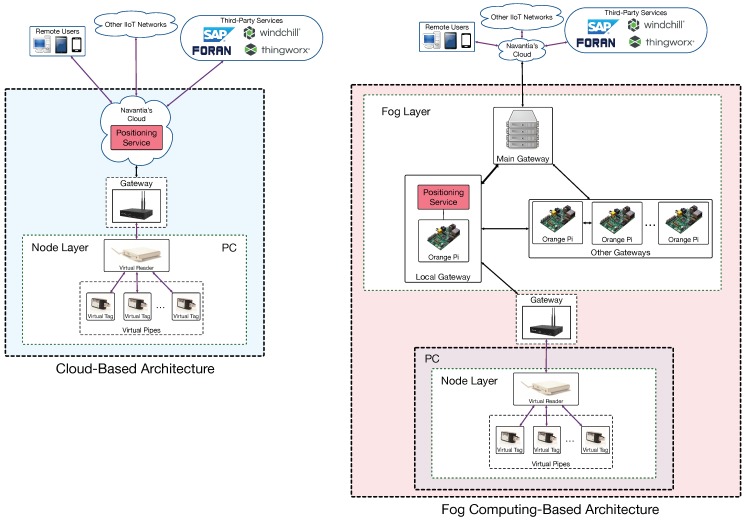
Implemented architectures.

Two scenarios were chosen to evaluate the proposed system. The first one was related to the cloud-based architecture depicted on the left of Figure 17. In such an architecture, the RFID tag information is collected by a gateway that transmits it to Navantia’s cloud, where it is processed by the positioning service. In the second scenario (on the right of Figure 17), RFID data are collected by local fog gateways that execute the positioning service.

In order to determine the performance of the system in both scenarios, their positioning service response latency was measured. Two sets of tests were performed. The first one was aimed at determining which system responded faster under regular loads (when managing less than 1000 tags). The goal of the second test was to find out the maximum number of supported tags. For such a purpose, both the cloud and the fog-based architectures were tested under abnormal high loads, when more than 10,000 tags sent information concurrently.

**Figure 18:**
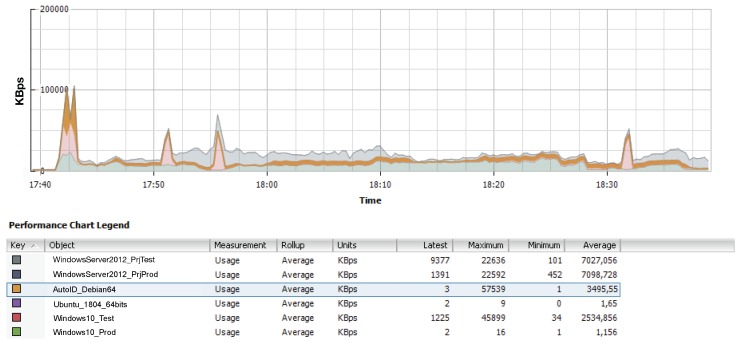
Network usage of the active virtual machines.

In the case of the cloud-based system, it is important to note that the virtual machine that executed the positioning service had no privileges over other virtual machines and it was not possible to fiddle with advanced high availability features to optimize response latency. Specifically, the cloud-based system shared its public network interface with another 11 virtual machines, although, during the tests, only six of them were active (i.e., they exchanged packets through the shared network interface). Figure 18 shows an ESXi stacked graph of the network usage of the active virtual machines. The area in orange is related to the network usage made by the virtual machine that runs the positioning service. It can be observed that the network load fluctuates through time and that there are certain time instants where relevant peaks exist (especially at the beginning of the graph, when the experiments were launched), which may impact network latency.

### 4.2. Latency and Processing Rate Under Regular Loads

The pipe workshop is considered to be under a regular load when up to 1000 pipes are being processed or stacked in the different areas. Thus, in order to evaluate the performance of the CPS under such a regular load, 10 different tests were performed when varying the number of tags from 200 to 1000 and when using a cloud-based or a fog-based architecture. For each test, one million SSI samples were generated.

**Figure 19:**
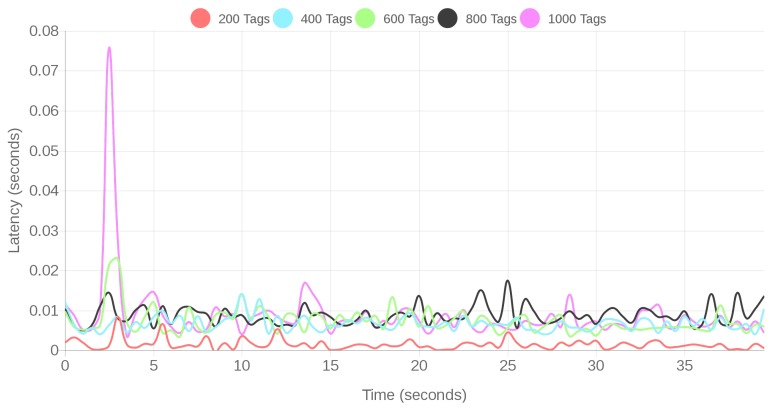
Latencies for the fog system under regular loads.

**Figure 20:**
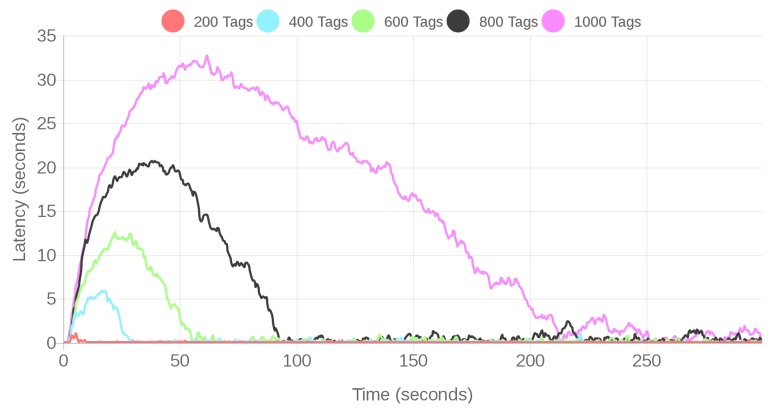
Latencies for the cloud system under regular loads.

The obtained latency results are presented in Figure 19 for the fog scenario and in Figure 20 for the cloud scenario. In both cases, a spike can be observed on the latency at the beginning of the tests due to the initial detection of the tags by the location subsystem. After the system gets stabilized, the latencies oscillate due to the random behavior of the virtual readers and tags (as explained in Section 4.1.2), but they do not increase as much as at the initial stage of every test. For the same number of tags, the latency obtained for the fog-based architecture is clearly smaller than for the cloud scenario. In the worst case (for 1000 tags), the maximum latency is 400 times larger than in the cloud-based architecture (roughly 32 s versus 0.08 s).

It can also be observed in Figure 20 that, for the cloud-based system, response latency increases with the number of tags monitored by the CPS. This behavior of the cloud-based system can be corroborated when calculating the average latencies, which are shown in Table 2. In contrast, the average latency for the fog computing system remains stable (in the order of milliseconds) despite the increase in the number of tags. It is important to indicate that the averages presented in Table 2 were calculated for the full time interval, including the initial spike. Note that such an interval varies from one scenario to another, since the stop condition consists in collecting one million SSI samples, which requires different amounts of time depending on the number of transmitting tags.

It is worth noting the field *Improvement* of Table 2, which indicates how many times the average latency of the cloud-based approach is larger than the respective latency of the fog-based architecture (it is shown that, on average, the fog responds between five and 481 times faster than the cloud). In addition, it can be observed that the increase in latency is not linear, what means that a cloud-based CPS would need much more hardware resources than a fog-based system when scaling it to manage more tags.

Despite the observed latencies, it can be concluded that, even in the worst case (for 1000 tags, cloud scenario), the latency is small (around 3.3 s on average) and should not influence the regular operation of the positioning service, which is not a real-time service (a slight delay is not critical for its operation). Nonetheless, it is interesting to measure the sample processing rate of the service in every scenario (i.e., the percentage of SSIs that are processed successfully by the positioning service). In such measurements it was obtained that, under regular loads, a processing rate of 99.99% was always achieved.

Note that it is not 100% because of how the number of received SSI samples is computed. Since the location subsystem is asynchronous, the received SSI samples are processed in batch by several parallel co-routines that end up obtaining an approximation of the number of samples. Such a number reaches a maximum difference of 10,000 samples with respect to the real value. Therefore, due to the fact that one million samples are processed, a maximum of a 1% error in the sample processing rate is introduced with this measurement technique.

### 4.3. Latency Under High Loads

The performance of the CPS can also be evaluated when it is under high loads, which would represent workshops with a remarkable density of monitored objects or a whole shipyard. Specifically, the performed high load tests made use of between 10,000 to 60,000 tags. Due to the large number of tags used, the total number of SSI samples was increased to 10 million to allow for receiving enough SSI samples from each tag.

**Figure 21:**
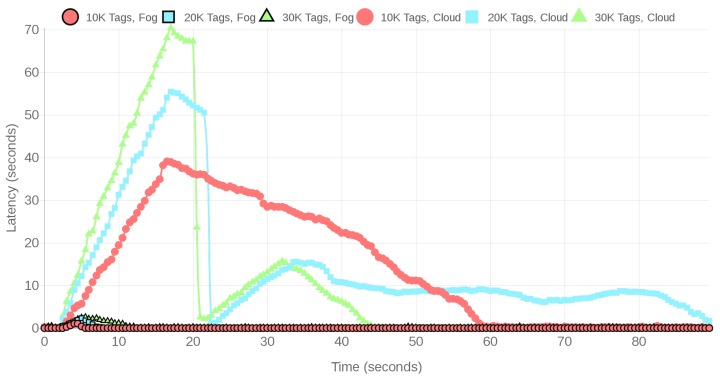
Latencies for the fog and cloud based systems under high loads.

**Figure 22:**
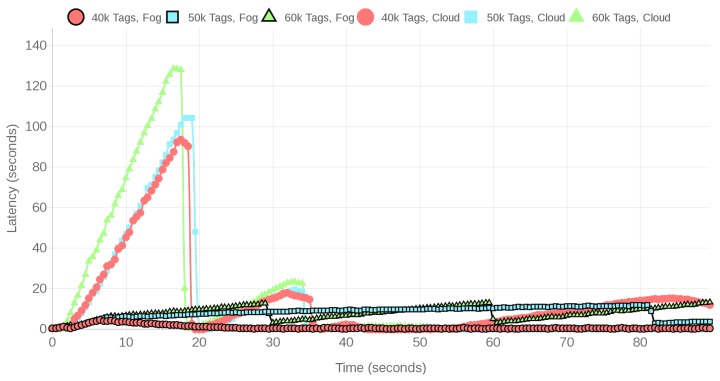
Latencies for the fog and cloud based systems under high loads.

Figure 21 and Figure 22 show the first time instants of the evolution of the positioning service latency when executed on the fog gateway and on the cloud. Like in the regular load scenario, the represented time interval was selected because it allows for observing the spike that occurs at the beginning of most tests because many tags start to transmit data within the first seconds. Then, after the spike, latency starts to decrease, increasing again only at certain time instants when a relevant number of tags transmit at the same time, impeding the location subsystem to process all the received tag SSI samples. This effect occurs periodically for every scenario, in a similar way as it can be observed clearly for the 60,000 tag curve for the fog system. In addition, as it is expected, latency increases as the number of concurrent tags goes up.

Table 3 compares the average latency responses for the fog gateway and the remote cloud server when 10,000 to 60,000 tags transmit data. Like in the regular-load scenario, the averages were calculated for the whole time interval of the experiment, which varies from one scenario to another, since the 10 million SSIs were collected faster or slower depending on the number of transmitting tags. Note that the average latencies should increase along with the number of tags, but, since the network and the communications interfaces are shared with other users, the final results oscillate slightly. Thus, the oscillations are not just a matter of averaging more samples, since measurements are conditioned by the actual network traffic conditions that may occur in a real-world cloud. However, such an oscillation has nothing to do with the fact that the fog gateway gets saturated for 50,000 tags due to its limited processing power. The cloud latency is also impacted by such an amount of tags, but it remains stable between 5 and 7 s. Nonetheless, it is important to note that is not realistic to try to manage more than 50,000 tags with a single fog gateway and that it would be straightforward to add more gateways to distribute the traffic load generated by the tags. In the case of the cloud, the system responds slower than the fog system for less than 50,000 tags, even when it is more powerful than the SBC, mainly because of the communications delays, which constitute a bottleneck under high traffic loads.

It is worth noting that, under regular network traffic conditions, the minimum/average/maximum round-trip times to the cloud are 47.25/49.3/62.87 ms, while the same times are 0.842/2.919/33.515 ms for the local fog gateway. However, despite such low round-trip times, for 10,000 tags the latency response of the cloud is almost 30 times slower than the average latency of the fog gateway. Nevertheless, it is important to emphasize that the obtained latencies can be decreased by varying different factors like the amount of network traffic that goes to the cloud, the physical distance from the RFID system to the cloud server, the cloud server computing power, or the load and speed of the devices that route the packets to the cloud.

Table 4 shows the sample processing rate for the fog and cloud-based solutions when processing samples from different amounts of virtual tags. The difference in performance between the fog and the cloud service is clear: although with 10,000 tags both fog and cloud systems obtain similar results, from 20,000 onwards, the cloud system gets overflowed by the traffic, so the number of processed samples falls dramatically. The same happens to the fog system after 50,000 tags, which indicates that more gateways or a more powerful gateway would be needed to handle such an amount of data.

**Figure 23:**
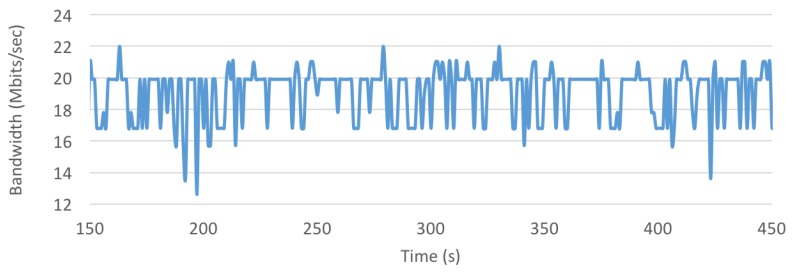
Bandwidth fluctuation when transmitting from an emulated tag to the cloud.

In the case of the cloud, for 20,000 and 50,000 tags, periodic high-load intervals occur because of sharing the system and network with other users, which impacted both the sample processing rates shown in Table 4 and the respective latencies included in Table 3. As was previously mentioned in Section 4.1.3, the fact of sharing cloud resources may impact the system performance, but it is not the only factor that affects the system response delay. In order to illustrate such an impact, Figure 23 shows how bandwidth (as measured by iPerf [56], which is the speed rate at which a sample file is transferred) fluctuates during a time interval due to the multiple user interactions through the network and routing/switching devices that exist in the network path that goes from the workshop where RFID readers are emulated until the data reaches the cloud. Although, for clarity, Figure 23 only represents a five-minute interval, bandwidth was measured with iPerf during more than six hours (almost a work shift). The results showed that bandwidth fluctuated constantly between 9.44 and 24.1 Mbps, although most of the time it remained stable within 17 and 20 Mbps (the overall average was 18.42 Mbps and the variance, 2.92 Mbps). Therefore, when designing a real-time positioning service like the one proposed in a cloud-based scenario, the entire path that the data will follow from the RFID readers until the cloud must be taken into account.

Finally, it is important to emphasize that, like in the latency tests, several factors may lower the sample processing rate, but there exist different alternatives to tackle the traffic load, like the use of load balancing or distributed computing.

### 4.4. Analysis of the Results and Key Findings

Since the different experiments performed are influenced by multiple factors (e.g., network infrastructure, network load, obstacles, emulated tag behavior), it is difficult to establish final conclusions, but the following are the most relevant key findings that would be preserved in similar industrial environments despite such deployment factors:

- When there is a regular number of monitored objects in a shipyard workshop (i.e., up to 1000), fog computing systems respond faster since they are closer to the data sources.
- For regular loads, the difference in response latency increases between the fog computing system and the cloud because of the amount of traffic exchanged, which collapses the cloud network progressively if no corrective measures are taken.
- In the shipyard, it is not usual to manage more than 1000 tags with only one computational device, but it is interesting to evaluate such a scenario to determine the performance of the implemented architectures. Thus, it can be observed that a single inexpensive fog gateway that runs the implemented positioning service can respond faster and with a higher sample processing success rate than a cloud-based system for up to 40,000 simultaneous tags. For 50,000 or more tags, the fog gateway gets saturated and the cloud system becomes a faster alternative, although its sample processing rate is lower than for the fog gateway.
- Response latency was the metric selected to measure the architecture performance in order to quantify user experience. Nevertheless, in certain scenarios, where it does not matter how fast the system updates its positions, other metrics could be used (e.g., deployment cost, power constraints, wireless range). Therefore, it is important to take the obtained results with caution since the system was designed explicitly according to Navantia’s requirements.
- Despite the observed under-performance of the cloud-based system, it is worth noting that it is possible to reduce the response latency by adjusting different high-availability parameters in VMWare ESXi [57]. Nevertheless, in terms of response latency, it seems that the proximity of the fog gateways is essential and, under regular loads, it would be difficult for the cloud to obtain lower round-trip times than the ones obtained by such devices.
- It is also fair to indicate that, during the experiments, every fog gateway only executed the positioning service, while, as it is illustrated in Figure 6, the fog-computing architecture was designed to provide multiple services on the same fog gateway. Therefore, it can be concluded that, although fog-computing services seem to be best option for providing low response latencies, their results may be influenced by the computational and network load associated with other services running simultaneously on the same fog gateway. However, note that, in a fog gateway, the number of concurrent service requests is actually low in comparison to the ones received by a cloud, since it only provides services to a reduced area (e.g., part of a workshop), while the cloud serves the whole company.
- It must be also noted that, during the tests, the behavior of the tags was emulated in order to evaluate the performance of the proposed architectures. In a real deployment, the results will differ due to the emulated tag behavior and because of the characteristics of the scenario (e.g., signal propagation, obstacles, presence of metal). Note also that, when several hundreds of tags respond at the same time, two main issues would arise:
  - Real tags implement a medium-access technique that was not emulated for the tests. Therefore, in a real deployment, individual tag delays will be higher than the ones obtained for the experiments, since tags have to synchronize their transmission intervals to avoid collisions.
  - In a real deployment there would be interference from other devices (the selected tags operate in an Industrial-Scientific-Medical (ISM) band) and from signals emitted asynchronously by other tags (e.g., signal reflections from distant tags), which, in practice, will increase collisions and will decrease the sample processing rate (because the actual SSI values would not be received at the positioning service).
- Finally, it is worth pointing out that the devised architecture, the technologies and the experiments discussed throughout this article were selected explicitly for a very hostile and specific industrial scenario, so the obtained results should not be generalized and each organization interested in deploying a similar system would have to adapt it to its own requirements.

## 5. Conclusions

Pipe traceability and tracking are essential in a shipyard due to their importance in shipbuilding. After analyzing the state of the art, this article presented the design and implementation of a CPS developed by Navantia and the University of A Coruña. The system is aimed at automating many of the tasks performed in a pipe workshop in order to accelerate production processes and increase the workshop output. Moreover, thanks to the use of a fog computing-based architecture, it is able to reduce latency, to provide location awareness and to cope with a large number of RFID tags.

In addition, the proposed CPS was integrated and tested successfully with Siemens’ MES (Simatic IT). Furthermore, it was evaluated in terms of latency response and sample processing rate, showing that, in the test scenarios, fog gateways respond faster than the cloud alternative and are also able to process successfully more samples under high traffic loads and are between five and 481 times faster under regular loads.

## Figures and Tables

**Table 1:** Characteristics of some of the latest and most relevant SBCs.

Name	Clock Rate	Cores	RAM	Cost (USD)
Banana Pi Pro [43]	1 GHz	2	1 GB	$ 55
BeagleBone Black [44]	1 GHz	1	512 MB	$ 56
Cubieboard 5 [45]	2 GHz	8	2 GB	$ 99
ODROID-XU4 [46]	2 GHz/1.4 GHz	8 (4 + 4)	2 GB	$ 59
Orange Pi PC [42]	1.6 GHz	4	1 GB	$ 15
Raspberry Pi 3 Model B+ [47]	1.4 GHz	4	1 GB	$ 35
UDOO X86 ULTRA [48]	2.56 GHz	4	8 GB	$ 267

**Table 2:** Fog and cloud average latencies under regular loads (in seconds).

Approach/#Tags	200	400	600	800	1000
Fog	0.0076	0.0023	0.0082	0.0073	0.0069
Cloud	0.0388	0.1369	0.3485	1.0978	3.3244
Improvement	×5.10	×59.52	×42.50	×150.38	×481.80

**Table 3:** Fog and cloud average latencies under high loads (in seconds).

Approach/#Tags	10,000	20,000	30,000	40,000	50,000	60,000
Fog	0.0234	0.055	0.116	0.295	8.467	7.756
Cloud	0.6899	2.027	1.339	2.980	7.406	5.834
Improvement	×29.48	×36.48	×11.54	×10.10	×0.87	×0.75

**Table 4:** Sample processing success rate under high loads.

Approach/#Tags	10,000	20,000	30,000	40,000	50,000	60,000
Fog	99.99 %	99.99 %	99.99 %	99.98 %	87.92 %	76.01%
Cloud	99.99 %	71.79 %	91.72 %	82.14 %	53.28 %	61.40 %

## Abbreviations

The following abbreviations are used in this manuscript:

API	Application Programming Interface
BI	Business Intelligence
CPS	Cyber-Physical System
ERP	Enterprise Resource Planning
FO	Fabrication Order
HF	High-Frequency
IAR	Industrial Augmented Reality
IoT	Internet of Things
ISM	Industrial-Scientific-Medical
MES	Manufacturing Execution System
PLC	Power-Line Communications
PLM	Product Life-cycle Management
RFID	Radio Frequency IDentification
REST	REpresentational State Transfer
SBC	Single Board Computer
SoC	System-on-Chip
SSI	Signal Strength Indicator
RSSI	Received Signal Strength Indicator
MES	Manufacturing Execution System
UHF	Ultra High Frequency
WSN	Wireless Sensor Networks
